# Focus on fatigue amongst young adults with spastic cerebral palsy

**DOI:** 10.1186/1743-0003-11-161

**Published:** 2014-12-11

**Authors:** Heleen A Russchen, Jorrit Slaman, Henk J Stam, Frederike van Markus-Doornbosch, Rita J van den Berg-Emons, Marij E Roebroeck, LEARN 2 MOVE Research Group

**Affiliations:** Department of Rehabilitation Medicine, Erasmus MC, University Medical Center, Rotterdam, the Netherlands; Sophia Rehabilitation, The Hague, The Netherlands; Rijndam Rehabilitation Institute, Rotterdam, The Netherlands

**Keywords:** Cerebral palsy, Fatigue, Physical activity, Cardiopulmonary fitness, Young adult

## Abstract

**Background:**

This study aimed to assess fatigue amongst young adults with spastic cerebral palsy (CP), to determine subgroups at risk for fatigue and to explore the relationship between fatigue and cardiopulmonary fitness and daily physical activity level.

**Participants:**

Young adults with spastic CP, Gross Motor Function Classification System (GMFCS) levels I to III, aged 16 to 24 years.

**Methods:**

Fatigue (Fatigue Severity Scale) and self-reported daily physical activity (Physical Activity Scale for Individuals with Physical Disabilities) were assessed for 56 participants using questionnaires. Daily physical activity was objectively measured using accelerometry (Vitamove system) over 72 hours. Progressive maximal aerobic cycling was used to measure cardiopulmonary fitness.

**Results:**

The mean Fatigue Severity Scale (FSS) score was 3.7 (SD 1.4). Forty percent of participants were fatigued, including 12.5% who were severely fatigued. Participants with bilateral CP (FSS = 4.2 (SD 1.4)) were more fatigued compared to those with unilateral CP (FSS = 3.1 (SD 1.3)) (p < 0.01). Levels of cardiopulmonary fitness (2.4 L/min (SD 0.8)) and daily physical activity (8.5% (SD 3.0)) were not significantly related to fatigue (respectively p = 0.10 and p = 0.55), although for cardiopulmonary fitness a trend was found.

**Conclusions:**

Fatigue is already present at a relatively young age amongst adults with CP, and CP subtype is a determinant of fatigue. We did not find significant evidence for a cross-sectional relation of fatigue with cardiopulmonary fitness or daily physical activity.

**Trial registration:**

Nederland’s trial register: NTR1785.

## Background

Cerebral palsy (CP) is a permanent disorder affecting the development of movement and posture that causes activity limitations and is attributed to non-progressive disturbances to the developing foetal or infant brain [1]. The prevalence of CP is high, at 1.5 to 3 per 1000 live births, and it is the most common physical disability amongst paediatric patients presenting to rehabilitation medicine [2]. The most frequently occurring CP type, the spastic form, is characterised by velocity-dependent increase in tonic stretch reflexes (‘muscle tone’) with exaggerated tendon jerks, resulting from hyperexcitability of the stretch reflex, as one component of the upper motor neuron syndrome [3].

In clinical practice, fatigue is reported frequently by both children and adults with CP. Nevertheless, only a few adult studies have sought to assess fatigue amongst persons with CP. One study showed that 61% of adults with bilateral CP were fatigued and 41% who were severely fatigued [4]. Another adult study of unilateral and bilateral CP showed that 30% were severely fatigued [5]. Thus, the prevalence of fatigue seems much higher amongst adults with CP compared to the general population, of which 18% report fatigue [4, 6, 7]. Research about fatigue in children with CP is scarce, but suggests that fatigue may greatly impact daily functioning [8]. Because of high fatigue levels amongst adults and children with CP [4, 5, 8, 9], fatigue is likely present amongst young adults with CP as well. However, systematic assessment of fatigue in young adults with CP has, to our knowledge, not been performed. Additionally, published studies have not addressed key factors to prevent or decrease fatigue amongst persons with CP.

Fatigue is a multifactorial symptom [4]. Fatigue can be defined as a subjective experience (the feeling of becoming easily tired, having a lack of energy or being exhausted); fatigue can also be defined as an objective experience (loss of strength in exercise). In the present study subjective fatigue is the end-point. It is hypothesised that fatigue in CP is related to personal characteristics (e.g. age and gender) and CP-related characteristics (e.g. level of gross motor function [10] and CP subtype (unilateral or bilateral)). In the present study, we focus on additional physical factors that may be related to fatigue: cardiopulmonary fitness and daily physical activity (PA) level. Durstine et al. have described a vicious circle of deconditioning [11] in which fatigue, low daily PA levels and low cardiopulmonary fitness influence each other. It is well established that both children and adults with CP have decreased levels of objectively measured daily PA [12–16] and cardiopulmonary fitness [17–21] compared to able-bodied age mates. Cardiopulmonary fitness is modifiable with rehabilitation [22]. Studies regarding the effectiveness of improving daily PA using lifestyle interventions seems inconsistent [23–25]. Evidence for the cycle of deconditioning amongst young adults and adults with CP is equivocal [5, 9, 17, 26, 27]; whereas a recent study suggest that exercise therapy helps to decrease fatigue amongst adults with CP [28]. Additional knowledge on correlates of fatigue in young adults with CP may yield information about specific subgroups at-risk and may provide starting points for treatment optimisation. During young adulthood, lifestyle interventions are expected to be appropriate to prepare for a healthy future. At this age, persons adopt to major changes in life occurring in their transition to adulthood and develop their adult lifestyle [29, 30].

The aim of the current study was to assess (severe) fatigue in young adults with spastic CP, and to investigate which subgroups are at increased risk for fatigue. Furthermore, we explored the relationship between fatigue and daily PA level and cardiopulmonary fitness, factors that are amenable to change.

## Methods

### Participants

Baseline data from the “Learn2Move 16-24” research project [23] were used for the current study. Participants were recruited from six rehabilitation centres and university hospital rehabilitation departments in the West-Central Netherlands and by the Association of Physically Disabled Persons and Their Parents (BOSK). Persons with CP were eligible if they were 16 to 24 years old and had a spastic form of CP that was classified as GMFCS level I (walks without restrictions) to III (walks with adaptive equipment assistance) [10]. Exclusion criteria were: disorders other than CP influencing daily PA level, contraindication to (maximal) exercise, severe cognitive impairment or insufficient comprehension of the Dutch language which would preclude understanding of the purpose of the project and its testing methods. An informational letter and invitation to participate were sent to eligible persons. A reminder was sent four weeks later to non-responders. All participants (and their parents in case of minority) provided written informed consent. The study was approved by the medical ethics committee of the Erasmus Medical Centre and local approval was granted by all participating centres.

### Fatigue

Fatigue was measured using the Dutch version of the Fatigue Severity Scale (FSS) [31, 32], a nine-item, self-administered questionnaire with scores ranging from 1 (strongly disagree) to 7 (strongly agree). The mean score ranges from 1 (no signs of fatigue) to 7 (most disabling fatigue). Fatigue is defined as an FSS score more than 1 standard deviation (SD) above the mean score for healthy Dutch persons (FSS ≥ 4.0) and severed fatigue is defined as a score of at least 2 SDs above the mean score for healthy Dutch persons (FSS ≥ 5.1) [32]. Internal consistency, reliability, validity and sensitivity of the FSS have been established in several patient groups [31, 32].

### Correlates of fatigue

Height was measured in the standing position. Body mass was measured whilst standing on an electronic scale and body mass index (BMI) (kg/m^2^) was calculated from height and body mass. Age, gender and CP subtype (unilateral or bilateral) were obtained from medical records. GMFCS level [10] was obtained from baseline measurements by the assessor.

### Daily physical activity

To objectively measure daily PA level, the Vitamove (VM) system (2M Engineering, Veldhoven, The Netherlands) was used. This system consists of three recorders applied to the trunk and thighs of ambulant participants. The non-ambulant participants were able to walk with or without walking aids, but use a wheelchair for long distances (GMFCS level II/III), used five recorders (trunk, thighs and wrists) to measure daily PA level. The VM system uses body fixed accelerometers which detect acceleration of the trunk, thighs and wrists. The VM system has demonstrated validity to quantify mobility-associated activities and detect inter-group differences in daily PA levels [33, 34]. Participants wore the VM system for 72 hours on randomly selected, consecutive weekdays. Measurements were analysed using VitaScore software (Vitascore BV, Gemert, The Netherlands). A detailed description of the activity detection procedure has been given elsewhere [33]. The duration of dynamic activities (composite measure consisting of walking, including climbing stairs and running; cycling; wheelchair propulsion, including hand cycling; and general noncyclic movement) was expressed as a percentage of a 24-hour period. Due to system failure or incorrect VitaMove system use, not all PA measurements lasted the entire 72 hours. Therefore, only VM system measurements covering at least 24 hours were used; eight participants’ data were excluded for this reason. Of the remaining participants (n = 48), six had at least a 24 hours of measurement, 24 had 48 hours of measurement and 18 had 72 hours of measurement.

To determine self-reported PA, the Physical Activity Scale for Individuals with Physical Disabilities (PASIPD) [35] was administered. This questionnaire consists of 12 items addressing leisure time, household activities, and work-related physical activities. We used the Dutch version of the PASIPD, which integrates lawn work and gardening into one item about gardening [36]. The total PASIPD score is obtained by multiplying the average hours per day for each item by a metabolic equivalent (MET) value associated with the intensity of the activity. The test-retest reliability of the PASIPD is good and the criterion validity is comparable with well-established, self-report PA questionnaires from the general population [36].

### Cardiopulmonary fitness

All participants could cycle; therefore, cardiopulmonary fitness was measured using a maximal ramp protocol on a cycle ergometer (Jaeger ER800 and Jaeger ER800SH, Jaeger Toennies, Breda, The Netherlands). The ergometry test began with a 3-minute warm-up without resistance. During the test, the resistance level was increased every 12 seconds by an amount that varied based on participant GMFCS level and gender. The target pedal rate was 60 to 70 rpm. The test concluded when participants stopped due to exhaustion or when participants were unable to maintain the initial pedal/crank rate. Gas exchange and heart rate (HR) were measured continuously during the test, using oximetry with two different, but comparable, breath-by-breath analysers (Oxycon Pro, ViaSys Healthcare, Houten, The Netherlands; Quark CPET system, Cosmed, Rome, Italy). These systems were calibrated prior to each measurement with reference gases of a known mixture. Cardiopulmonary fitness was defined as the highest mean oxygen uptake during 30 seconds of exercise (VO2peak in L/min). Subjective strain was measured immediately after the final stage by the Borg Scale for Rating of Perceived Exercise [37]. Prior to testing, all participants were screened by a rehabilitation physician and contraindications to PA were assessed using the Physical Activity Readiness Questionnaire (PARQ) [38]. Forty-four persons met objective criteria for maximal exercise: a peak heart rate of at least 90% of the predicted maximum heart rate of 194 beats per minute [39] or a respiratory exchange ratio greater than or equal to 1.1 [40]. For these 44 participants cardiopulmonary fitness levels were compared to healthy norm-values using the formula of Jones et al., based on gender, height, age and weight [41]. Persons who performed a valid maximal exercise test did not differ from those excluded on this criterion (n = 12) for age, gender, CP subtype, GMFCS level, BMI (p > 0.05), or mean FSS-scores (3.5 versus 4.2; p = 0.14).

### Statistical analysis

Descriptive statistics were used to evaluate fatigue level, proportion of (severely) fatigued participants, personal and CP-specific characteristics, daily PA and cardiopulmonary fitness. Because data were normally distributed, we applied parametric statistical tests. We determined that persons who performed valid maximal exercise tests (n = 44) did not differ from those excluded on this criterion, using the independent sample *t*-test (age), Chi-square tests (gender, CP subtype, GMFCS level) and the Mann–Whitney *U* test (BMI). VO2peak was compared with healthy norm values using paired samples t-tests [41]. Univariate linear regression analyses were used to evaluate whether fatigue was related to personal or CP-related characteristics or with modifiable factors: daily PA or cardiopulmonary fitness. A multivariate linear regression model was used to correct for age, gender, GMFCS level, BMI and CP subtype, as these variables confound the relationships between fatigue and modifiable factors (change of more than 10% in β). GMFCS levels were included in the analyses as dichotomous variables. Due to the low number of participants classified as GMFCS level III, participants with GMFCS level II and III were combined into one category. When significant relationships were observed between fatigue and modifiable factors, subgroup analyses were performed. Independent sample t-tests were used to test the difference in fatigue level; Chi square-tests were used to test for differences in proportion of (severely) fatigued participants. SPSS Inc. (Released 2008. SPSS Statistics for Windows, Version 17.0. Chicago: SPSS Inc.) was used. A P value of ≤ 0.05 was considered statistically significant. A P value of 0.05 - 0.10 was considered as a trend, but does not reach the statistically significant threshold.

## Results

### Participants

Fifty-six young adults, with a mean age of 20 (SD 2.8) years were included in this study. Five participants used wheelchairs in daily life for long distances but were able to walk with or without walking aids (GMFCS level II/III). Participant characteristics are presented in Table 1.

**Table 1:** **Personal and cerebral palsy-related characteristics, daily physical activity and cardiopulmonary fitness**

**Personal and CP-related factors**	
Age, in years (SD)	20.0 (±2.8)
Males/Females	27/29
GMFCS level, I/II/III	31/21/4
BMI, in kg/m^2^ (SD)	23.3 (±4.8)
CP subtype, unilateral/bilateral	29/26^a^
**Physical activity and cardiopulmonary fitness**	
% dynamic activities (SD), *(n = 48)* ^b^	8.5 (±3.0)
PASIPD, MET hrs/day (SD)^c,^ *(n = 53)* ^d^	12.8 (±8.3)
VO2peak, in L/min (SD), *(n = 44)* ^*e*^	2.4 (±0.8)
VO2peak, in mL/min/kg (SD), *(n = 44)* ^*e*^	34.9 (±9.1)

^a^CP subtype data was missing for one participant.

^b^Eight participants did not have measurements of at least 24 hours.

^c^Percentage of time per 24 hours spent walking, cycling, using wheelchair propulsion or in general noncyclic movement.

^d^The PASIPD was not performed by three participants.

^e^Twelve participants did not meet criteria for maximal exercise testing.

CP = cerebral palsy; GMFCS = Gross Motor Function Classification System; BMI = Body Mass Index; PASIPD = Physical Activity Scale for Individuals with Physical Disabilities.

### Fatigue

Fatigue scores are presented in Table 2. The mean FSS score was 3.7 (SD 1.4, range 1.1 to 6.8). Of all participants, 39.3% were fatigued, including 12.5% who were severely fatigued.

**Table 2:** **Fatigue and comparison between unilateral and bilateral cerebral palsy**

	Total (n = 56)	Unilateral CP (n = 29)	Bilateral CP (n = 26)	P ^a^
FSS score, mean (SD)	3.7 (±1.4)	3.1 (±1.3)	4.2 (±1.4)	<0.01^b^
FSS ≥4.0, n (%)	22 (39.3)	10 (34.4)	12 (46.2)	0.38
FSS ≥5.1, n (%)	7 (12.5)	1 (3.4)	6 (23.1)	0.03^b^

^a^P values for independent sample t-tests (FSS score) or Chi square-test (proportion of fatigue) for differences between unilateral and bilateral CP groups are presented.

^b^Significant (p ≤ 0.05) difference between unilateral and bilateral CP groups.

CP subtype was missing for one participant.

CP = cerebral palsy; FSS = Fatigue Severity Scale.

### Correlates of fatigue

Table 3 shows the relationships between fatigue level and personal and CP-related factors, as well as physical activity and cardiopulmonary fitness. Persons with bilateral CP (FSS = 4.2 (SD 1.4)) were more fatigued compared to those with unilateral CP (FSS = 3.1 (SD 1.3)) (p < 0.01). The proportion of severely fatigued participants was also higher in the bilateral CP group (23.1%) compared to the unilateral group (3.4%) (p = 0.03) (Table 2). The univariate regression analysis showed a trend between fatigue and GMFCS level (p = 0.08). Age, gender and BMI were not related significantly to fatigue.

**Table 3:** **Personal, cerebral palsy-related, physical activity and cardiopulmonary fitness correlates of fatigue**

Correlate	β uncorrected	95% CI for β	P	β corrected ^a^	95% CI for β	P
Age	0.12	(-0.14, 0.39)	0.37			
Gender^b^	-0.20	(-0.46, 0.06)	0.13			
GMFCS level^b^	0.23	(-0.03, 0.49)	0.08			
BMI	0.00	(-0.22, 0.22)	0.99			
CP subtype^b^	0.39	(0.14, 0.64)	<0.01			
% dynamic activities^c^	0.03	(-0.26, 0.31)	0.86	0.08	(-0.19, 0.35)	0.55
PASIPD	-0.07	(-0.35, 0.20)	0.62	0.01	(-0.24, 0.26)	0.92
VO2peak	-0.29	(-0.58, 0.00)	0.06	-0.27	(-0.59, 0.04)	0.10

^a^Correlates are corrected for age, gender, GMFCS-level, BMI and CP subtype, as these variables confounded the relationships (change of more than 10% in β).

^b^Categorical correlates were coded in the following manner: gender: female = 0, male = 1; GMFCS-level: GMFCS-level 1 = 0, GMFCS-level 2 and 3 = 1; CP subtype: unilateral = 0, bilateral = 1.

^c^Percentage of time per 24 hours spent in dynamic activities.

CP = cerebral palsy; GMFCS = Gross Motor Function Classification System; PASIPD = Physical Activity Scale for Individuals with Physical Disabilities; BMI = Body Mass Index.

Objectively measured daily PA level was 8.5% (SD 3.0), which represents 123 minutes of PA per day (Table 1). The mean PASIPD score for participants who completed the questionnaire was 12.8 (SD 8.3) MET hrs/day. Objective and self-reported daily PA were not related to fatigue (Table 3). PA level has been described elsewhere into more detail [16].

The mean VO2peak score of the 44 participants who met criteria for maximal exercise was 2.4 (SD 0.8) L/min, which is 15% lower (p < 0.01) compared to calculated healthy norm-values for cardiopulmonary fitness [41]. The mean maximum load was 173 (±69) W. The mean Borg-scale score was 6.5 (heavy to very heavy exercise). Other aspects of cardiopulmonary fitness have been described elsewhere [21]. The regression analysis of fatigue and cardiopulmonary fitness showed a trend toward an association (p = 0.06) that persisted after correction for confounders (p = 0.10) (Table 3).

## Discussion

### Fatigue

Fatigue is already present at a relatively young age amongst adults with CP. The mean fatigue score of FSS = 3.6 was relatively high as compared to a healthy reference population (FSS = 3.0) [6, 32]. Although the reference values for fatigue refer to an adult population within a broader age-range, we think that these may be appropriately compared to the present sample of young adults with CP since fatigue and age seemed unrelated within this age-range, according to Valko et al. [6] The high fatigue levels observed amongst young adults with CP may suggest that at this age fatigue should be checked as a potential health issue to address.

### Subgroups at increased risk

Fatigue level seems to be more affected by CP-related characteristics than personal characteristics. Participants with bilateral CP were more fatigued and should be considered at risk for higher levels of fatigue. Also participants with GMFCS level II or III seemed to be more fatigued compared to those with GMFCS level I, although this relationship did not reach significance. The mean fatigue level for bilaterally affected persons was comparable to that found in a previous study amongst adults with bilateral CP [4]. A possible explanation for the difference in fatigue between persons with bilateral and unilateral CP may be a difference in physical strain. Persons with bilateral CP are likely to have a higher strain during walking, which could lead to more fatigue [42]. Unlike the general population [6], gender was not related to fatigue in young adults with CP. Considering the potential role of overweight and obesity for fatigue in persons with CP, waist circumference might be a more sensitive parameter as compared with BMI [21].

### Fatigue related to daily physical activity or cardiopulmonary fitness

From a rehabilitation perspective, focusing on daily PA and cardiopulmonary fitness is of interest, with assumed ameliorating effects on other health issues such as fatigue. Similar to adults with CP, the young adults in this study had low daily PA levels [17, 43]. In addition, the lack of a relationship between daily PA and fatigue is consistent with previous studies of adults with CP [5, 9, 17]. It is known that, because of high levels of physical strain during walking, persons with CP may decrease their daily PA levels to conserve energy or prevent fatigue [42]. One could hypothesize that this compensation strategy may explain the absence of a relationship between daily PA and fatigue in persons with CP. Also the low levels of cardiopulmonary fitness compared to healthy reference values are consistent with previous research in children, adolescents and adults with spastic CP [17, 19, 20]. Although we found a trend towards lower fatigue in persons with higher cardiopulmonary fitness, we did not find significant evidence for a cross-sectional relation between fatigue and cardiopulmonary fitness or daily PA. In contrast, taking deterioration in walking function into account, Opheim et al. found a relationship between this parameter and fatigue in adults with CP, thus hypothesizing that fatigue in adults with CP was of physical origin [9]. Contrasting results are also seen in longitudinal data from the present sample, showing evidence for mediating effects of PA levels and cardiopulmonary fitness on a decrease of fatigue severity [27]. Although in those analyses fatigue was measured with a different questionnaire, namely the fatigue subscale of the Checklist Individual Strength (CIS-f), these conflicting results may also reflect the importance of studying relationships between parameters longitudinally, in order to adequately assess the vicious circle of deconditioning [11]. Thus, to better understand the role of associated factors for fatigue in persons with CP, we may broaden our focus by addressing longitudinal relationships and by including other factors besides daily PA and cardiopulmonary fitness. An example of other health factors that may play a role in fatigue are sleep disorders, for which persons with CP are known to be at an increased risk [44]. Also, behavioural factors, stress, depression, pain or medication may influence fatigue. Therefore, rehabilitation programmes that aim to decrease fatigue levels should choose a multifactorial approach.

### Study limitations

A limitation of this study is the lack of a control group. Fatigue levels are compared with healthy reference populations and not with a age-matched control group. Reference measurements are possibly done under different circumstances, so a reliable comparison is difficult. Another limitation of this study is the use of questionnaires to assess fatigue and the possibility of recall. However, no alternatives exists to assess experienced fatigue. In addition, we may have overestimated daily PA and cardiopulmonary fitness because of selection bias. Persons who are interested in cardiopulmonary fitness and daily PA may have higher levels of these parameters and may be more likely to participate in the study. Beside this interest bias, persons with GMFCS levels I and II were overrepresented in our study population, which may have resulted in overestimation of levels of cardiopulmonary fitness and daily PA. Nevertheless, daily PA levels and cardiopulmonary fitness were still subnormal in this study. We also excluded participants performing submaximal exercise during cardiopulmonary fitness measurements, with the remaining participants may have overestimated cardiopulmonary fitness level. Because personal or CP-related characteristics and FSS scores for the excluded group were comparable to those of included persons, selection bias seems to be unlikely in this area. Thus, with some caution our results are generalisable to young adults with CP without severe cognitive impairment. Finally, the cross-sectional design of this part of the study precludes any determinations about time course or cause-effect relationships of fatigue with other factors.

## Conclusions

Fatigue is a common problem amongst young adults with spastic CP, with almost 40% experiencing fatigue. Participants with bilateral CP are especially at risk for high levels of fatigue. Cross-sectional relationships of fatigue with cardiopulmonary fitness or daily PA were not significant, although a trend was seen that persons with a higher cardiopulmonary fitness were less fatigued. Based on these results, we recommend that rehabilitation programmes to decrease fatigue for young adults with CP should use a multifactorial approach.

## Abbreviations

CP: Cerebral palsy
PA: Physical activity
GMFCS: Gross motor function classification system
PASIPD: Physical activity scale for individuals with physical disabilities
BMI: Body mass index
FSS: Fatigue severity scale.
